# Infection after the use of INFIX in Pelvic Ring Injuries

**DOI:** 10.1051/sicotj/2021047

**Published:** 2021-09-08

**Authors:** Rahul Vaidya, Karun Amar, Derrek Woodbury, Austen Washington

**Affiliations:** 1 Detroit Medical Center, 5th Floor Heart Hospital 311 Mack Avenue Detroit Michigan 48201 USA

**Keywords:** Infection, Infix, Irrigation, Debridement, Pelvic, Ring

## Abstract

*Introduction*: The purpose of this study is to report on infection with anterior subcutaneous internal pelvic fixation (INFIX) for pelvic ring injuries and the outcomes of treatment. *Methods*: An IRB-approved retrospective study was performed using trauma databases of a level one and level two trauma center from 2012–2018. Infection after the INFIX procedure was diagnosed in 10 of 179 cases. Treatment included formal irrigation and debridement, removal of the hardware, and culture-specific antibiotics. Patients were followed for a minimum of 12 months. Recorded outcomes include X-rays, Majeed scores, and the presence of any loss of reduction using reduction parameters. *Results*: Time to detect the infection was 54.2 ± 24.3 days (range 24–90, median 56 days). *Staphylococcus aureus* was the most common bacteria isolated. The average follow-up was 830 ± 170 days (range 575–1088 days). All patients went on to the radiographic union. There were no recurrent infections or osteomyelitis at the latest follow-up. Patients maintained their reduction after INFIX removal (KI), and Majeed scores ranged from 72 to 96 (seven good, three excellent). *Discussion*: Infections after using the INFIX procedure were dealt with by irrigating and debriding the wounds, removing the INFIX with culture-specific antibiotics for 2–6 weeks. Implants were maintained for at least 25 days, and there was no loss of reduction. There were no long-term sequelae noted in this small series or the literature review included in this paper.

## Introduction

The use of an anterior subcutaneous internal fixator (INFIX) has been reported in the literature to reduce and fix unstable pelvic ring injuries with the appropriate posterior fixation [1–19]. Most authors report the INFIX is well tolerated by patients, allowing good mobility and outcomes. The downside is the need for secondary surgery to remove the implants, recommended three months post-op or later [20]. A recent systematic review reports heterotopic ossification (36%), lateral femoral cutaneous nerve (LFCN) irritation (26.3%), infection, and femoral nerve palsy (1%) as some of the possible complications. The incidence of infection after INFIX is reported as 1–3% [1–19]. Due to the limited numbers of infected cases in the INFIX series, there is no current recommendation on treatment. The purpose of this paper is to report on infections after the use of INFIX for pelvic ring injuries, report on outcomes and review the literature. We also asked if there is a risk of loss of reduction by removing the INFIX early.

## Materials and methods

An IRB-approved retrospective study was performed using trauma databases of one level one and one level two trauma center. We retrospectively reviewed all patients from July 2012 to December 2018 who underwent INFIX and appropriate posterior fixation. The indication for this index surgery was an unstable pelvic fracture in which the surgeon believed that there was a need for anterior fixation. At the time of index fixation, all the patients underwent supplemental percutaneous sacroiliac screw fixation or open fixation of the posterior pelvis plus lumbopelvic fixation when indicated. Per the Surgical Care Improvement Project guidelines, all patients received preoperative antibiotics before the start of surgery.

Among this group of patients, 10 of 179 (5.6%) were subsequently diagnosed with infection and were included in this study. There were five women and five men with an average age of 37.4 years (range, 14–67 years) and a follow-up of 830 ± 170 days (range 575–1088 days). Two of ten infected cases also had supplemental lumbopelvic fixation.

**Figure 1:**
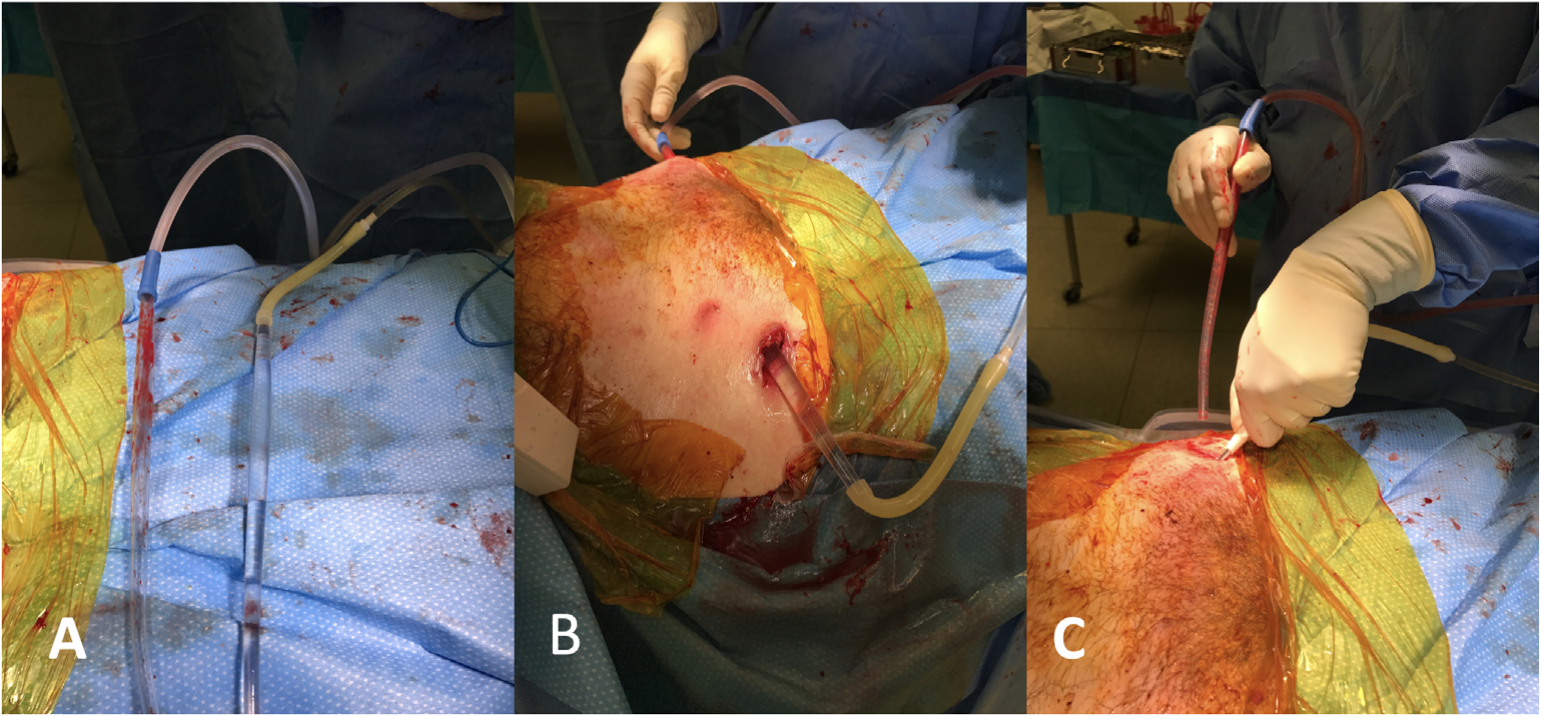
(a) After removing the implants, the wounds were washed with 6–9 L of Normal Saline with cystoscopy tubing and a plastic Yankour tip. (b) For the subcutaneous tunnel of the bar, on one side, the saline flowed in, and on the other, we used suction. (c) The pin sites were also curetted in the bone and washed with smaller tips fastened to the cystoscopy tubing.

The following information was recorded: age, gender, presence of comorbidities, Young/Burgess fracture classifications [21], date of initial surgery, date of implant removal, length of follow-up, method of injury, associated injuries, and culture-specific antibiotic. (Table 1) Positive cultures from a draining wound determined presence of infection. Upon detection, patients were taken to the operating room for formal irrigation and debridement (I&D) and hardware removal. Irrigation and debridement was performed with deep cultures taken from pin sites and the area of the subcutaneous bar. The skin incisions were not expanded from the original insertion procedure. We washed with 6–9 L of Normal Saline with cystoscopy tubing and a Yankauer tip (Figure 1), curetted the pin sites, and removed the INFIX. The Yankauer suctions were used to instill irrigation and suction it out from the subcutaneous tunnel (Refer Supplementary Video). After the I&D, culture-specific antibiotics were recommended and administered by the infectious disease team with close follow-up in the clinic. Standard pelvic imaging series consisting of anterior posterior (AP) and inlet/outlet views were obtained in the clinic, and the radiographic union was assessed. Patients were followed for a minimum of 12 months in the clinic with X-rays. Patients were invited back for a final visit for this study where an exam, X-rays, and clinical outcome score as described by Majeed [22] was performed. Loss of reduction was measured using the modified Keshishyan Index [23, 24].

**Table 1:** Patient demographics.

Patient	Age	Sex	BMI	Class	Other pelvic fixation	Bug isolated	# of washout	follow-up days
1	26	F	21.7	LC3	R S2 SI × 2	*Strep. agalactiae*	1	783
2	45	M	27.2	LC1	R S1 SI, R S2 SI	MSSA	1	975
3	67	M	22.4	LC3	R S1 SI, L S1 SI	*Enterobacter cloacae*	2	977
4	57	M	17	LC1	R S1 SI, L S1 SI Bilat lumbopelvic fixation	MSSA	1	1088
5	53	F	36	LC2	R S1 SI, R S2 SI, Plates	Corynebacterium, Staphylococcus epidermidis, *Proteus mirabilis*	1	840
6	26	M	25.2	LC2	R S1 SI	MRSA	1	905
7	21	F	24.7	LC 3	R Lumbopelvic, R S1 TSS	MSSA	1	881
8	28	F	24.3	APC2/APC3	Left S1 SI, S2 TSS	MRSA	2	605
9	14	M	42.3	APC3/APC2	Bilateral S1 SI	*Enterobacter cloacae*	2	575
10	87	F	24	LC2	L 2 LC2 Screws	MSSA	1	672

S1 or S2 = sacral body segment; SI, sacroiliac screw; TSS, Trans sacral screw; LC2 = Lateral compression two screw; MRSA, methicillin-resistant *Staphylococcus aureus*; MSSA, methicillin-sensitive *Staphylococcus aureus*; RS1 SI = Right Sacral 1st body sacroiliac screw.

## Results

**Figure 2:**
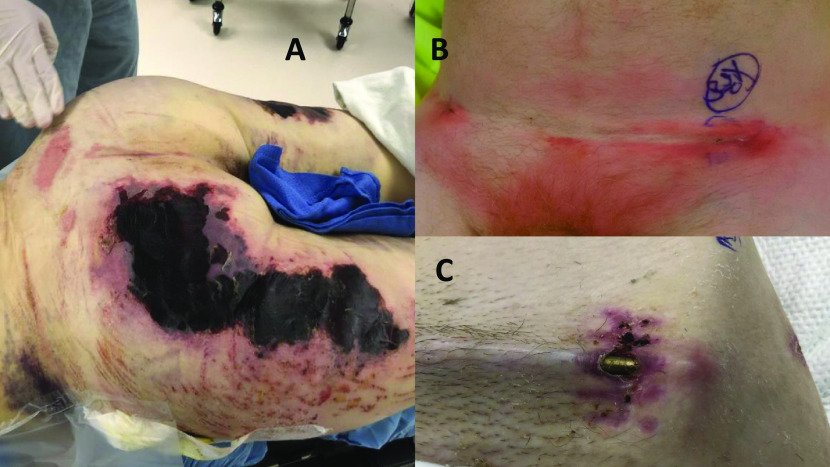
Clinical pictures of (a) a case of a large Morel-Lavallée lesion, (b) draining wound with exposed implants, (c) a draining wound and cellulitis.

**Figure 3:**
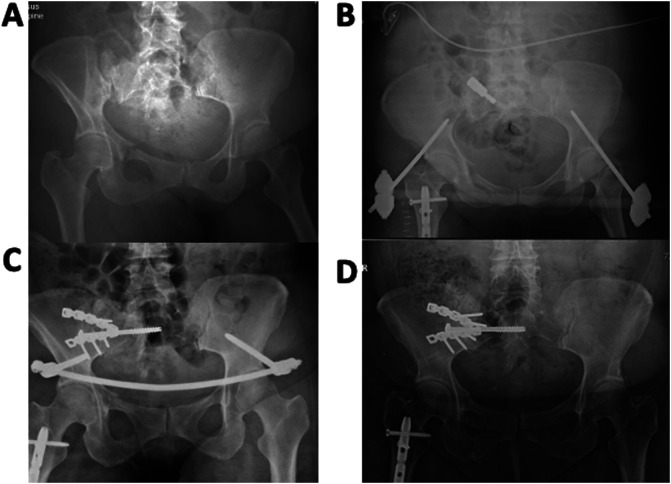
In one case, a supra-acetabular external fixator was used, and the resulting INFIX got infected despite using different holes for the implants. (a) Preop X-ray, (b) with temporary external fixator, (c) after definitive fixation, (d) at last follow-up.

The infection rate was 10/179 (5.6%), which is slightly higher than reported in the literature (see Table 3). Risk factors included: One patient with insulin-dependent diabetes mellitus, one had hepatitis C liver cirrhosis and ascites, two were active smokers, one had a history of intravenous drug abuse, and three had a significant Morel-Lavallée lesion (Figure 2). One patient had a prior supraacetabular external fixator for two days, but different holes were used for the INFIX. (Figure 3 case example). Due to the small number of patients, we could not attribute any significantly increased risk from these variables compared to the noninfected cases.

Motor Vehicle Collision was the most common mechanism of injury (6/10), followed by fall from height (2/10), and pedestrian versus auto (2/10). All were closed injuries. There were two Young and Burgess LC1 injuries, two LC2 injuries, three LC3 injuries, one APC2 injury, and two APC3 injuries (seven lateral compression injuries vs. three anterior posterior compression injuries).

Time to detect infection ranged from 24 to 90 days from the index procedure, with an average of 54.2 ± 24.3 days and a median of 56 days. In five cases, the infection was detected within 30 days, two cases between 30 and 60 days, and three cases between 60 and 90 days. In nine cases, the patient was taken to the OR within two days, and in one case, suppressive antibiotics were tried for 14 days which failed to suppress the drainage. The patient was then taken to the OR for formal I and D and removal of the implant.

Three patients had a second I and D procedure during this hospitalization and seven had a single procedure. The second procedure in three cases was that there was persistent drainage after the first washout.

There was no loss of reduction for any case due to explant as measured by the modified Keshishyan Index [24]. The earliest we removed implants was 25 days.

*Staphylococcus aureus* was the most common bacteria isolated and was found in 6/10 patients. The other species identified included *Enterobacter cloacae* in two patients, *Staphylococcus epidermidis* in one, and *Streptococcus agalactiae* in the final patient (Table 2).

**Table 2:** Infection isolation and treatment data.

	Bug isolated	Hospital Abx	Discharge Abx	Out pt tx
1	*Strep. agalactiae*	Vancomycin iv	Clindamycin po	6 weeks
2	MSSA	Sulfamethoxazole & trimethoprim double strength po	Sulfamethoxazole & trimethoprim double strength po	2 weeks
3	*Enterobacter cloacae*	Ertapenem iv	Ciprofloxacin po	2 weeks
4	MSSA	Ceftriaxone iv	Clindamycin po	4 weeks
5	Corynebacterium, Staphylococcus epidermidis, *Proteus mirabilis*	Clindamycin iv	Clindamycin po	2 weeks
6	MRSA	Vancomycin iv	Vancomycin iv	6 weeks
7	MSSA	Vancomycin iv	Sulfamethoxazole & trimethoprim double strength po	2 weeks
8	MRSA	Vancomycin iv	Sulfamethoxazole & trimethoprim double strength po	2 weeks
9	*Enterobacter cloacae*	Ertapenem iv	Ciprofloxacin po	2 weeks
10	MSSA	Cefazolin iv	Cefazolin iv	2 weeks

MRSA, methicillin-resistant *Staphylococcus aureus*; MSSA, methicillin sensitive *Staphylococcus aureus*; iv, intravenous; po, oral (par os).

All patients had an infectious disease consult who recommended a culture-specific antibiotic regimen (Table 2) that continued as an out-patient for two weeks in seven patients, four weeks in one patient, and six weeks in two patients. This was based on the Infectious Disease Physicians recommendation, and unfortunately, it led to a varied duration of treatment. The length of stay for these patients due to the infection was 7.5 days ± 5.4 range 1–18 days in nine patients. One patient had a three-month length stay from his original injury unrelated to the pelvic fracture or INFIX infection. The infection was treated during his extended stay for abdominal injuries.

All the patients went on to radiographic union as determined by radiographs obtained in clinic and the ability to weight bear as tolerated with minimal pain. None of the patients developed recurrent infections at the INFIX site or any sign of osteomyelitis thus far.

Total Majeed scores for the patients ranged from 72 to 96, with an average score of 81.7 at the latest follow-up. When converting these raw scores to functional status, all ten patients had final grading of good or excellent (seven rated as good and three as excellent).

## Discussion

The use of INFIX in pelvic ring injuries has been adopted and reported from many centers globally [20]. What to do when an infection with this procedure has not been well delineated as it is uncommon (3%) [20]. The rate of INFIX infection in this series was 5.6% (10/179). The diagnosis was made between three weeks and three months, it was based on drainage from the wounds and was confirmed with positive cultures. Risk factors included diabetes (1), liver cirrhosis (1), smoking (2), IV drug abuser (1), and a significant Morel-Lavallée lesion (3). We could not establish any significant associations or risk factors with these low numbers compared to the overall group. One patient had a prior supraacetabular external fixator for two days, and when we placed the INFIX, we drilled new holes for the pedicle screws, but this did not prevent an infection from occurring. In our practice, if we are placing an emergency anterior external fixator, we try to use iliac crest pins so that if we use an INFIX for definitive fixation, the supra acetabular sites are clean. All patients had the implant removed at 25 days or later post-surgery, and no loss of reduction was noted. Seven patients had a single washout following our protocol, and three had two washouts related to persistent drainage from the wound. We used a Yankauer tip to thoroughly irrigate the subcutaneous tract and the supra-acetabular pin sites, which were also curetted. This seems to work, but there is no comparison procedure as this was what we did, and it was not based on prior knowledge in these cases. Antibiotics were given based on the organism isolated. Intravenous antibiotics were given in the hospital, and then outpatient antibiotics were prescribed for two weeks (seven patients), four weeks (one patient), and six weeks (two patients) after discharge based on the Infectious Disease team’s recommendation. That did not make any difference in the long-term outcome. No patient suffered a second infection after removal of the implants, and there was no instance of osteomyelitis during the follow-up period, which averaged 830 ± 170 days. All these pelvic fractures healed. This was based on x-rays which showed healing and the ability to weight bear without difficulty or loss of reduction.

The limitations of this study and topic are that there are only ten cases of INFIX infection in this study and only 14 others in the literature, so it is difficult to make solid recommendations. This is simply a description of what was done and the outcome.

In a recent systemic review on the use of INFIX in pelvic injuries, infection at the surgical site was reported in 15 of 496 cases (3%) [19]. In 9/15 cases, the infection occurred before 10 weeks, and in 3/15 cases, it occurred late after six months (three cases not elaborated). The implant was removed in ten cases, followed by culture-specific antibiotics. In two early cases, the infection was treated with culture-specific suppressive antibiotics, and it resolved to allow for explantation at the usual time. Infections resolved in all cases with no reports of osteomyelitis at the latest follow-up (Table 3).

**Table 3:** Infix infection literature review.

Infection	Number	Early, <10 wks	Late	Explant	Kept	Total cases
Vaidya et al. [3, 4]	3	3		3		91
Merriman et al. [6]	1		1	1		3
Müller et al. [7]	2	2		1	1	36
Hoskins et al. [10]	3	NR		NR	NR	21
Fang et al. [12]	1	1		1		29
Shetty et al. [17]	1	1			1	15
Dahill et al. [18]	1		1	1		47
Steer et al. [20]	2	2		1	1	24
Current Study	10	7	3	10		179
Total	24	16	5	18	3	445

In the current series, antibiotic suppression of the infection was attempted in one case to delay the removal of the implant. This did not work as there was continued drainage, and eventually, the hardware was removed two weeks later. The patient tolerated the draining wound.

From these cases and review of the literature, it seems that having the INFIX in for at least four weeks is adequate to maintain the overall reduction in the face of infection. We have also wondered that if the INFIX is exposed, is an absolute indication for removal. In several cases with massive pelvic wounds and soft tissue coverage issues, the INFIX has been utilized as a low elevation external fixator or exposed on one side while being covered on the opposite side. Due to its low profile, it is easy to cover the implant, and the wounds with a wound vacuum-assisted closure device (vac). In these cases, this also did not lead to osteomyelitis and was tolerated by the patients [25]. There have been reports of using suppressive antibiotics in the face of INFIX infection in several patients until the 3-month mark, and we feel that probably four weeks is adequate to remove the implant as most pelvic fractures get sticky at this time [19]. We had no loss of reduction when the infix was removed after 25 days.

## Conclusion

The primary method of treatment of infections after using the INFIX involved irrigating and debriding the wounds, removing the INFIX implant after a minimum of four weeks and treating with 2–4 weeks of culture-specific antibiotics. In early infections (< 4 weeks, two reported in the literature), the choice seems to be to irrigate, debride, treat with suppressive antibiotics and hopefully remove the implant after four weeks. There were no long-term sequelae that were noted in this series or the literature review.

## Conflicts of interest

RV owns patents on Anterior Subcutaneous Pelvic Fixation / the INFIX Procedure, which have never restricted any use of the procedure and are available for use. The remaining authors have no relevant conflicts of interest to declare that could jeopardize the validity of the content of this article.

## Funding

No funding was received to assist with the preparation of this manuscript.

## Ethics approval

The study was approved by the IRB of our home institution.

## Informed consent

Written or verbal informed consent was obtained from all patients included in the study.

## Authors’ contributions

K. Amar and D. Woodbury: data gathering; R. Vaidya: drafting the manuscript, data presentation, and data gathering; A. Washington: manuscript preparation, formatting, and editing.

## Supplementary Material

Supplementary material is available at https://www.sicot-j.org/10.1051/sicotj/2021047/olm. *Video S1*: Using the Yankauer tip to irrigate the subcutaneous tunnel.
